# Heat transport in inclined flow towards a rotating disk under MHD

**DOI:** 10.1038/s41598-023-32828-6

**Published:** 2023-04-12

**Authors:** K. Mahmud, Faisal Z. Duraihem, R. Mehmood, S. Sarkar, S. Saleem

**Affiliations:** 1 Department of Mathematics, Faculty of Natural Sciences, HITEC University Taxila Cantt, Taxila, 47070 Pakistan; 2 Department of Mathematics, College of Sciences, King Saud University, P.O. Box 2455, Riyadh, Saudi Arabia; 3 School of Applied Sciences (Mathematics), Kalinga Institute of Industrial Technology Bhubaneswar, Bhubaneswar, India; 4 Department of Mathematics, King Khalid University, Abha, Saudi Arabia

**Keywords:** Mechanical engineering, Mathematics and computing

## Abstract

Flow towards a rotating disk is of highly practical significance in numerous engineering applications such as Turbine disks, rotary type machine systems and many more. In light of this, the current work is an attempt to explore MHD oblique flow towards a rotating disk. Hydromagnetic effects in addition to heat transfer is taken into consideration. The flow governing Partial Differential Equations are altered to a system to coupled non-linear Ordinary Differential Equations through scaling group of transformations which afterwards are tackled using Shooting Algorithm. The impact of obliqueness parameter *γ*, rotation ratio parameter $\alpha$ and magnetic field parameter M on 2-dimensional and 3-dimensional stream contours are presented. Location of the shear center varies with magnetic field parameter. Heat flow at the disk surface boosts with magnet field parameter M and rotation ratio parameter $\alpha$.

## Introduction

Flow towards a rotating disk is a subject of hot research for modern day scientists, engineers and mathematicians. Natural examples of rotating flows as Spiral galaxies, hurricanes, Circulations in the oceans and also in our daily lives such as vortex in bath-tub and vortex generated in a tea-cup by stirring. Rotating flows, on the other hand, are more than just observations. Literature survey reveals that pioneer inquiry of the flow over a limitless rotating disk was done by Kármán et al.^1^. He used traditional transformations for similarity to alter the subject into a system of ordinary differential equations. Afterwards numerous researchers were motivated to discuss several engineering and industrial problems related with the rotating flow with different effects. Attia et al.^2^ investigated variations in rotating flow under the varying magnetic field on rotating flow. Von Kármán’s investigation the flow towards a rotating disk by using a fluid with a high viscosity, was further discussed by Miklavčič^3^. He studied how a rough rotating disk change the flow and observed that disk surface allows partial slip. For uniform roughness, azimuthal velocityl decreases as slip increases. For Von Kármán’s problem i.e., for the case of no-slip condition, initially the radial velocity is zero then attains a maximum value and then reduces to zero.

Alam et al.^4^ considered rotating disc to discuss the decomposition of micro sized particals in conjunction with convective heat transfer and observed that the velocity of axial thermophoresis is enhanced by raising the thermophoresis parameter values, the coefficient of thermophoresis, unsteadiness parameter and rotational parameter. Wang^5^ assumed non-aligned stagnation point flow for rotating disc and found that torque remains constant but makes the flow field and surface shear more complicated. Shevchuk et al.^6^ developed an integral method to discuss heat transfer for rotating disk and compared the results obtained by this method are in agreement with those obtained by exact solution. It has been found that solely the unique characteristics of the impinging jet determine heat transmission. The threshold value of thermal conductivity is practically independent of Prandtl number. Munawar et al.^7^ supposed different physical properties by taking an axisymmetric stagnation point flow into account of rotating disc. There are practical problems of rotating disc where orthogonal flow is not consistent. The situations exist where a fluid towards a rotating disc is not orthogonal.

Niimi et al.^8^ examined the three dimensional axisymmetric oblique flow on a plane wall around the stagnation point and observed that the wall stress only depends upon the distance from the stagnation point and shear stress is zero at the point of stagnation and origin is the point where stress becomes maximum. Rahimi et al.^9^ considered non-orthogonal three dimensional flow towards a moving cylinder to investigate variations of different rotatory parameters on velocity profiles and noticed that shear stress increases due to the increase in Reynolds number and suction rate. Sabzevar et al.^10^ assumed the flow with certain strain rate impinging on plate non-orthogonally with transpiration. He concluded that with the change of transpiration rate there is a huge impact on the distribution of the velocity components, pressure and temperature and also observed that the stagnation point slighted away from the origin due to the effect of free stream obliqueness.

Many novel uses for magnetic fluid have evolved in recent years, including inertial dampers, rotating shaft seals, loudspeakers, gauges, and sensors. Many uses of magnetic fluid rely on their inherent qualities, such as their capacity to conduct magnetic flux, levitate magnetic or non-magnetic objects, and many more^11^. The analysis of MHD fluid flow towards a rotating disk has received a lot of interest through recent research in the literature. This is because rotating disk flows of conducting fluids are very important since they have both theoretical and practical applications in a wide range of fields, such as rotating machinery, thermal power generating systems, computer storage devices, medical equipment, air cleaning machines, gas turbine rotors, techniques for crystal formation and, most notably, aerodynamic uses. The magnetic properties and behavior of electrically conducting fluids are investigated in magneto hydrodynamics.This phenomena is using the concept that an electric current is generated by applying a magnetic field to a fluid. A reciprocal force that causes a change in magnetic field is Lorentz force. Magnetohydrodynamics offers many different applications in the fields of biomedical sciences and designing. Tumors treatment, division of cells, blood streams, tissue temperatures, and MHD control plants are applications of Magnetohydrodynamics. Three dimensional MHD stagnation flow due to a stretchable rotating disk was investigated by Mustafa^12^. It is concluded that shear stresses and stagnation velocities are very dependent upon the rotation parameter, magnetic field parameter, and stretching parameter. The near disc radial velocity drastically increases with an increase in radial stretching, and this is balanced by a drop in the azimuthal and axial velocities. For minor disk rotation, small stretching initially causes an increase in radial velocity, which is followed by a steady reduction with significant stretching. All the parameters are found to cause an increase in rotational shear stress, which in turn causes an increase in torque.The radial shear stress rises in the case of small stretching for small rotation values, however this trend is inverted for big rotation. Li et al.^13^ proposed a brand-new magneto-rheological fluid brake concept and examined it both theoretically and numerically with heat dissipation, especially when only performing emergency brakes. On the basis of modified Bingham model, apparent viscosity as a parameter was adapted to depict the magnetic field’s relationship, temperature field, and flow field. The results showed that the design is dependent on intensity distribution of magnetic flux induction and also simulations are consistent with the experimental results. These results are very helpful for the structure design, optimization, and upgrading the products related to magneto-rheological fluid. He extended the earlier analysis on fluid-solid coupling analyses.

Widodo et al.^14^ applied numerical technique, Keller box, to system of equations developed for Magnetohydrodynamics nanofluids into a porous cylinder. He discussed the results by considering variation of Prandtl number, magnetic parameter, volume fraction, and porosity parameter. These results showed that velocity profile is directly related and temperature profile is inversely related to magnetic parameter and also porosity parameter.

Mustafa et al.^15^ extended Von Kármán problem related to infinite rotating rough surface disk when there is a nanofluid that conducts electricity. It is shown that velocity distributions and magnetic field parameter are inversely proportional to each other and also heat flux at a rotating disk has no effect due to Brownian motion. Ramzan et al.^16^ considered micropolar nanofluid moving in the direction of a rotating disk with the condition of presence of magnetic field and also condition of partial slip is considered. It is observed that velocity and spin component are decreased by slip effect and magnetic field. Mahanthesh et al.^17^ assumed uneven conditions for the heat source and heat flux for the research of MHD flow of nano liquids caused by a rotating disk. Soid et al.^18^ discussed flow toward a disc that is radially contracting and expanding. It is concluded that magnetic parameter and skin friction coefficient both are directly proportional to each other. Rudy et al.^19^ explored flow of partial slip over a rotating disk by using porous medium with condition of magnetohydrodynamics boundary layer along combined with chemical reaction and thermal radiation. It is observed that the relationship between the temperature profiles and the nano particle volume fraction parameter are directly proportional to each other. MHD fluid flow towards a rotating disk by considering effects of homogeneous-heterogeneous reactions are discussed by Gholinia et al.^20^. Zangooee et al.^21^investigated flow for both homogeneous and heterogeneous reactions in the presence of a magnetic field between two stretchable and rotating disks. Reynolds number and stretching parameters on temperature, concentration, radial, axial, and tangential velocities are taken under consideration and results elaborates that with increasing the values of stretching rate of lower disk, velocities increases in the vicinity of lower disk. Temperature and concentration also rise as the lower disk’s stretching rate does and vice versa. It is highlighted that concentration and temperature behaviors are opposite.

Mandala et al.^22^ investigated the entropy analysis of nanofluid towards a rotating porous disk with consideration of magnetic field. Three-dimensional hydromagnetic entropy analysis of a biviscosity nanofluid moving at a time-dependent stretching rate in the direction of the disk’s radius as it approaches a rotating porous disk reveals that the fluid is accelerated as the disk’s rotation speed increases in both the radial and cross-radial axes. At disk, Heat transfer rate and skin-friction are dependent on thermal radiation, disk rotation, porous medium permeability and nanosize particles.Entropy of the system is defined by Bejan. Mehdi et al.^23^ considered MHD flow between two disks that are rotating in presence of the effects of nanomaterials and thermal radiation. It is elaborated that tangential velocity and temperature distribution decreases for increasing value of nanoparticles and thermal radiation.

Heat transfer phenomena is very advantageous and applicable in many fields like technological processes, engineering, and industries. The creation of energy by some inexpensive means is a critical, renewable and crucial component of a country’s industrial development. Heat transmission in MHD ferrofluid flow towards a rotating disk which is stretchable was explored by Mustafa et al.^24^. When three distinct types of ferroparticles are considered, the results show that as parameter volume fraction is increased, radial velocity falls when azimuthal velocity and temperature increase and also due to thermal conductivity and maximum density, skin friction factor in the radial direction and also local Nusselt number of magnetite ferrofluid increases. Turkyilmazoglu et al.^25^ analysed transfer of heat due to nanofluid towards a rotating disk andIt is emphasised that heat transfer is much enhanced by addition of nanofluid. Very similar tendencies are seen for all nanofluids in the temperature field, such as an increase in the volume percentage of nanoparticles increasing the creation of heat and the thickness of the thermal layer increased as a result and oppositely temperature gradient at the wall is lowered. The heat transfer rate is linearly increased despite the Nusselt number’s multiplying factor. Usman et al.^26^ investigated heat transmission from a tangent hyperbolic fluid to a stretchable rotating disc with a lubricating surface. He noticed that as the Weissenberg number and slip value at the interface grow, so does the velocity profile. Some recent related studies can be found in Nayak et al.^27^, Siddra et al.^28^ and Tabassum et al.^29^.

Sarkar et al.^30^ studied the inclined stagnation point flow above a rotating disc. For the first time, he used the appropriate similarity transformations to convert the governing mass and momentum conservation equations into a set of ordinary differential equations. In the presence of nonlinear Rosseland thermal radiation, Qasim et al.^31^ studied the two-dimensional boundary layer flow of Jeffrey fluid across a radially extending disk and by lowering the variable viscosity ratio parameter, a reduction in fluid velocity and the thickness of the momentum boundary layer is observed. Shah et al.^32^ investigated the impact of pulsatile pressure gradient on irregular blood flow via an inclined tapering cylindrical tube of porous media in the presence of a transverse magnetic field. He noticed that there is a sizable variation between the flow’s velocities at later times when the Reynolds number is small and large. Nazeer et al.^33^ discussed Magnetohydrodynamics (MHD) of a non-Newtonian fluid with heat transfer through a porous medium and observed that Newtonian multiphase fluid is less common than Jeffrey multiphase suspension.

Gangadhar et al.^34^ discused the convective heat transfer characteristics of a hybrid nanofluid mixture with slip effects by considering Tiwari-Das nanofluid modeland and he concluded that increased slip effects greatly enhance the local heat transfer rate in the nodal and saddle point regions. Some very recent related studies can be found in^35–44^. Sharma et al.^45^ discussed the transfer of heat over a porous rotating disk and concluded that with the increase in the magnetic field, viscous dissipation, and the thermal radiation mechanism, the magnitude of the magnetic-fluid temperature is also increased. We may find some related studies in^46,47^.

Flow of a fluid over rotating disk has many applications in engineering and industry. Rotating bodies, centrifugal pumps, viscometers, rotors, fans, turbines, spinning disks are examples of rotating disk flows. Other than its fundamental importance, the study is relevant for many practical applications such as cooling of silicon wafers, rotating blades and chemical processes.

There are some investigations where it is assumed an axisymmetric stagnation point flow aligned with the axis of the rotating disc. However, this assumption is not consistent with every practical situation. There may also be situations where a fluid is obliquely impinging on a rotating disc at an arbitrary angle of incidence.

The detailed literature review of above cited work reveals that although flow towards a rotating disk has been a topic of keen interest for researchers, scientists and engineers owing to its vital significance in several practical industrial applications, yet there is no contribution in the literature regarding heat transfer analysis on oblique flow towards a rotating disk. We aim to discuss this problem in due detail.

The structure of the paper is formulated as follows. Section “Introduction” comprises of introduction and literature survey. In Section “Mathematical formulation” mathematical modeling of the proposed problem is presented in detail. Numerical solution and physical discussion of the obtained results is presented in Section “Numerical results and discussion”. Finally concluding remarks are presented in Section “Concluding remarks”.

## Mathematical formulation

**Figure 1:**
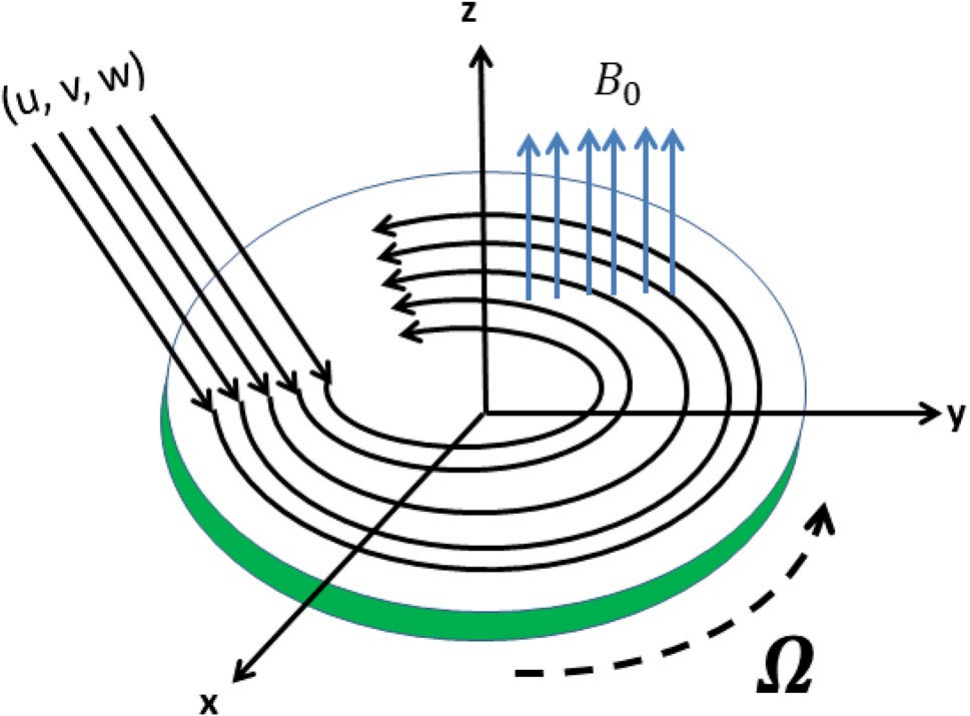
Problem’s geometry.

We have considered three dimensional steady viscous fluid flow which impinges diagonally on an infinite dimensional rotating disk placed along $xy-$plane. The symbol used for angular velocity is $\Omega$ which is considered constant and region of the fluid is taken as $z > 0$. The coordinate system is fixed. Using Cartesian coordinates (*x*, *y*, *z*) with x-, y- axes on the disk and z-axis normal to the disk and also (*u*, *v*, *w*) are components of velocity which are taken along the coordinates (*x*, *y*, *z*) define the problem’s geometry and is shown in Fig. 1.

Consider the flow field as given in^8^.

1 $$u_{e} = ax + bz^{{\frac{1}{2}}} ,v_{e} = ay,w_{e} = - 2az,T = T_{\infty } atz \to \infty$$

In the above equation *a* represents the strain rate related to flow of irrotational stagnation point and the strain rate related to shear flow is denoted by *b*. The shear flow is taken along x-axis. Under the above conditions, following is the resulted system of equations

2 $$\begin{aligned} \frac{\partial u}{\partial x}+\frac{\partial v}{\partial y}+\frac{\partial w}{\partial z}&=0, \end{aligned}$$

3 $$\begin{aligned} u\frac{\partial u}{\partial x}+v\frac{\partial u}{\partial y}+w\frac{\partial u}{\partial z}&=\nu \nabla ^2u-\frac{1}{\rho }\frac{\partial P}{\partial x}-\frac{\sigma B_0^2 u}{\rho }, \end{aligned}$$

4 $$\begin{aligned} u\frac{\partial v}{\partial x}+v\frac{\partial v}{\partial y}+w\frac{\partial v}{\partial z}&=\nu \nabla ^2v-\frac{1}{\rho }\frac{\partial P}{\partial y} -\frac{\sigma B_0^2 v}{\rho }, \end{aligned}$$

5 $$\begin{aligned} u\frac{\partial w}{\partial x}+v\frac{\partial w}{\partial y}+w\frac{\partial w}{\partial z}&=\nu \nabla ^2w-\frac{1}{\rho }\frac{\partial P}{\partial z},\end{aligned}$$

6 $$\begin{aligned} u\frac{\partial T}{\partial x}+v\frac{\partial T}{\partial y}+w\frac{\partial T}{\partial z}&=\alpha \nabla ^2T. \end{aligned}$$

where $\nu$, $\rho$, *P*, and T are the kinematic viscosity, the density, the pressure, and temperature respectively. The condition on the disc,

7 $$\begin{aligned} u=-\Omega y, v=-\Omega x, w=0, T=T_{w} \text {at} z=0 \end{aligned}$$

For large z, Eqs. (2)–(6) reduce to

8 $$\begin{aligned} -\frac{1}{\rho }\frac{\partial P}{\partial x}&=u_e\frac{\partial u_e}{\partial x}+w_e\frac{\partial u_e}{\partial z}+\frac{\sigma B_0^2 u_e}{\rho }, \end{aligned}$$

9 $$\begin{aligned} -\frac{1}{\rho }\frac{\partial P}{\partial y}&=v_e\frac{\partial v_e}{\partial y}+\frac{\sigma B_0^2 v_e}{\rho }, \end{aligned}$$

10 $$\begin{aligned} -\frac{1}{\rho }\frac{\partial P}{\partial z}&=w_e\frac{\partial w_e}{\partial z}, \end{aligned}$$

11 $$\begin{aligned} w\frac{\partial T}{\partial z}&=\alpha \frac{\partial ^2T}{\partial z^2}. \end{aligned}$$

which convert governing momentum conservation laws and heat transfer into:

12 $$\begin{aligned} \frac{\partial u}{\partial x}+\frac{\partial v}{\partial y}+\frac{\partial w}{\partial z}&=0, \end{aligned}$$

13 $$\begin{aligned} u\frac{\partial u}{\partial x}+v\frac{\partial u}{\partial y}+w\frac{\partial u}{\partial z}&=u_e\frac{\partial u_e}{\partial x}+w_e\frac{\partial u_e}{\partial z}+\nu \nabla ^2u+\frac{\sigma B_0^2 u_e}{\rho }-\frac{\sigma B_0^2 u}{\rho }, \end{aligned}$$

14 $$\begin{aligned} u\frac{\partial v}{\partial x}+v\frac{\partial v}{\partial y}+w\frac{\partial v}{\partial z}&=v_e\frac{\partial v_e}{\partial y}+\nu \nabla ^2v+\frac{\sigma B_0^2 v_e}{\rho } -\frac{\sigma B_0^2 v}{\rho }, \end{aligned}$$

15 $$\begin{aligned} u\frac{\partial w}{\partial x}+v\frac{\partial w}{\partial y}+w\frac{\partial w}{\partial z}&=w_e\frac{\partial w_e}{\partial z}+\nu \nabla ^2w, \end{aligned}$$

16 $$\begin{aligned} w\frac{\partial T}{\partial z}&=\alpha \frac{\partial ^2T}{\partial z^2}. \end{aligned}$$

For disk is not rotating i.e., $\Omega = 0$ the above system of equations then reduced into flow of axisymmetric oblique stagnation point.

By exploiting the similarity techniques, resulting highly non-linear partial differential equations (PDEs) are converted into system of ODEs. This method is very useful for solving such type of PDE arising in the problems of boundary layer flows. We have used the similarity transformations^30^ as given below

17 $$\begin{aligned} \left. \begin{array}{ll} u&{}=axf^{\prime }(\zeta )-\Omega yg(\zeta )+\sqrt{a\nu }h(\zeta ),\\ v&{}=ayf^{\prime }(\zeta )-\Omega xg(\zeta ),\\ w&{}=-2\sqrt{a\nu }f(\zeta ),\\ \theta (\zeta )&{}=\frac{T-T_{\infty }}{T_{w}-T_{\infty }}; \zeta =\sqrt{\frac{a}{\nu }}z. \end{array}\right\} \end{aligned}$$

Using these similarity variables into (13)–(16), the PDEs are altered into system of ordinary differential equations (ODEs):

18 $$\begin{aligned} f^{\prime \prime \prime }(\zeta )+2 f(\zeta )f^{\prime \prime }(\zeta )-M^2f^{\prime }(\zeta )-f^{\prime 2}(\zeta )+M^2+1+\alpha ^2 (g(\zeta ))^2=0, \end{aligned}$$

19 $$\begin{aligned} g^{\prime \prime }(\zeta )+2 f(\zeta )g^{\prime }(\zeta )-2g(\zeta )f^{\prime }(\zeta )-M^2g(\zeta )=0, \end{aligned}$$

20 $$\begin{aligned} h^{\prime \prime }(\zeta )+2 f(\zeta )h^{\prime }(\zeta )-M^2h(\zeta )+M^2\gamma \zeta ^{\frac{1}{2}}-h(\zeta )f^{\prime }(\zeta )=0, \end{aligned}$$

21 $$\begin{aligned} \theta ^{\prime \prime }(\zeta )+2 Pr f(\zeta ) \theta ^{\prime }(\zeta )=0, \end{aligned}$$

and we obtain the following boundary conditions

22 $$\begin{aligned} f(\zeta )=0, g(\zeta )=1, f^{\prime }(\zeta )=0, h(\zeta )=0,\theta (\zeta )=1 \text {at} \zeta =0, \end{aligned}$$

23 $$\begin{aligned} f(\zeta )=\zeta , h(\zeta )=\gamma \zeta ^{\frac{1}{2}}, g^{\prime }(\zeta )=0,\theta (\zeta )=0 \text {at} \zeta \rightarrow \infty . \end{aligned}$$

where $\alpha =\frac{\Omega }{a}$ is a parameter known as rotational ratio parameter and $\gamma =\frac{b}{\sqrt{a}}\frac{1}{(a\nu )^{\frac{1}{4}}}$ denotes the obliqueness parameter and $M^2=\frac{\sigma B_0^2}{\rho a}$ is the magnetic flux parameter. When $\alpha =0$ in Eqs. (18)–(21) and also in its boundary conditions (22) and (23) represent the equations of flow on wall when flow is of axisymmetric non-orthogonal stagnation point. By reducing b = 0, Eqs. (18)–(21) along with its boundary conditions (22) and (23) are the equations of flow towards a rotating disc when stagnation point flow is of axisymmetric orthogonal. Wall shear stress components are defined as

24 $$\begin{aligned} \tau _{\chi }=\rho \nu \frac{\partial u}{\partial z}|_{z=0} = a \rho \nu [{\chi } f^{\prime \prime }(0)-\alpha {\eta } g^{\prime }(0)+ h^{\prime }(0)], \end{aligned}$$

25 $$\begin{aligned} \tau _{\eta }=\rho \nu \frac{\partial v}{\partial z}|_{z=0} =a \rho \nu [{\eta } f^{\prime \prime }(0)+\alpha {\chi } g^{\prime }(0)]. \end{aligned}$$

By solving $\tau _{\chi }=0$ and $\tau _{\eta }=0$, we obtained shear center where shear is zero. From Eqs. (24) and (25),

26 $$\begin{aligned} \chi _s=-\frac{f^{\prime \prime }(0) h^{\prime }(0)}{(f^{\prime \prime }(0))^2+\alpha ^2 (g^{\prime }(0))^2}, \eta _s=-\frac{ \alpha h^{\prime }(0)g^{\prime }(0)}{(f^{\prime \prime }(0))^2+\alpha ^2 (g^{\prime }(0))^2}. \end{aligned}$$

and at the disk surface, the temperature gradient is

27 $$\begin{aligned} -\frac{\partial T}{\partial y}|_{y=0}=-\theta ^{\prime }(0). \end{aligned}$$

## Numerical results and discussion

The system of non-linear Eqs. (18)–(21) having boundary conditions (22)–(23) are numerically resolved using the MATLAB software’s bvp4c approach. In Shampine et al.^48^, the bvp4c method is described in full. Heat transfer and flow characteristics of the modeled physical problem are examined against various physical factors such as magnetic flux parameter *M*, rotation ratio parameter $\alpha$, obliqueness parameter $\gamma$ and prandtl number $\Pr$. A suitable agreement in the numerical values is obtained with the results of Wang^5^ in Table 1.

**Table 1:** $f^{\prime \prime }(0)$ and $g^{\prime }(0)$ for various $\alpha$ when $M=0$.

$\alpha$	$f^{\prime \prime }(0)$		$g^{\prime }(0)$	
	Present	Wang^5^	Present	Wang^5^
0	1.311937694	1.31194	$-$ 1.074669925	$-$ 1.07467
1	1.573920484	1.57539	$-$ 1.10999972	$-$ 1.1100
2	2.295642287	2.2951	$-$ 1.196830974	$-$ 1.1968
3	3.365654471	3.3657	$-$ 1.305521615	$-$ 1.3055
5	6.259875006	6.2602	$-$ 1.531982498	$-$ 1.5320
7	9.916516906	9.9165	$-$ 1.745105685	$-$ 1.7451
10	16.522814801	16.5229	$-$ 2.033027951	$-$ 2.0330

**Figure 2:**
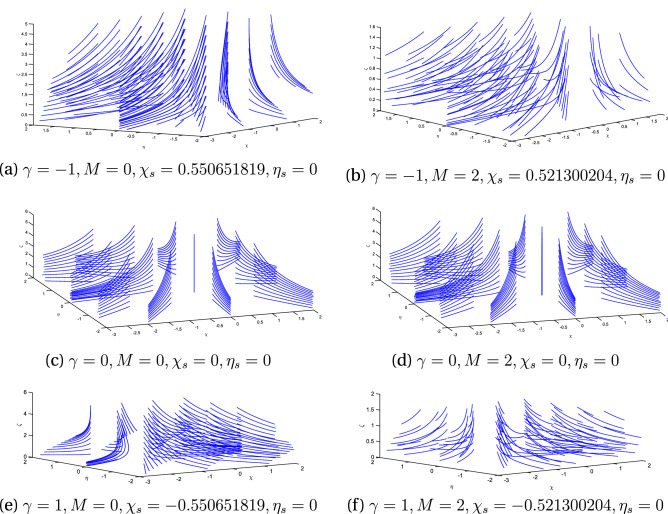
Streamlines for $\alpha =0$ with variation in oblique parameter $\gamma$ and magnetic parameter M.

**Figure 3:**
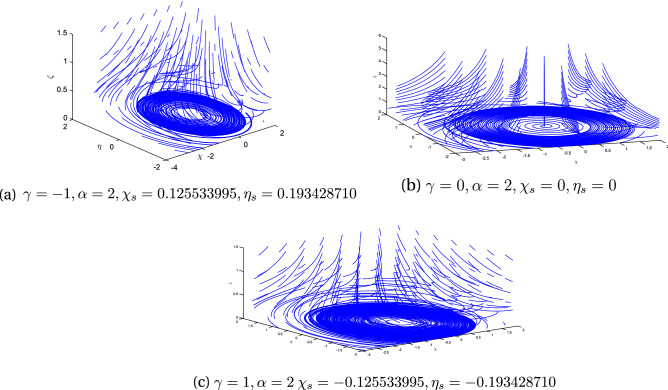
Streamlines for $M=2$ with variation in rotation parameter $\alpha$ and obliqueness parameter $\gamma .$.

**Figure 4:**
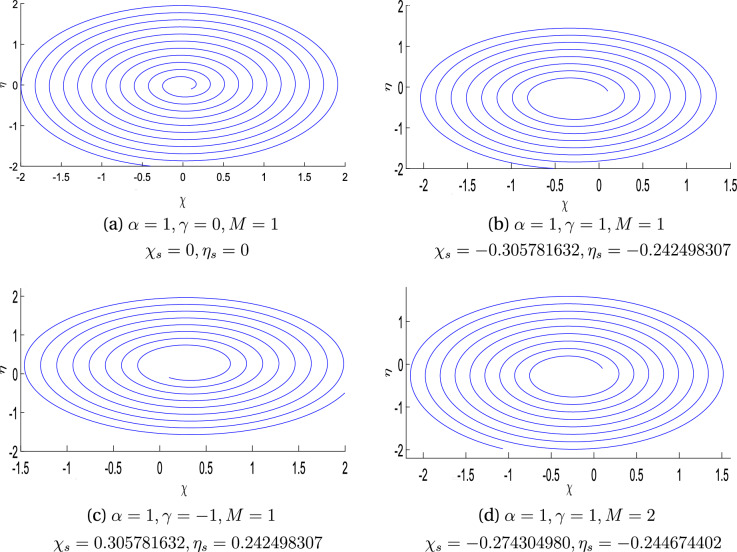
Contours for $\alpha =1$ with the variation of obliqueness parameter $\gamma$ and magnetic parameter M.

**Figure 5:**
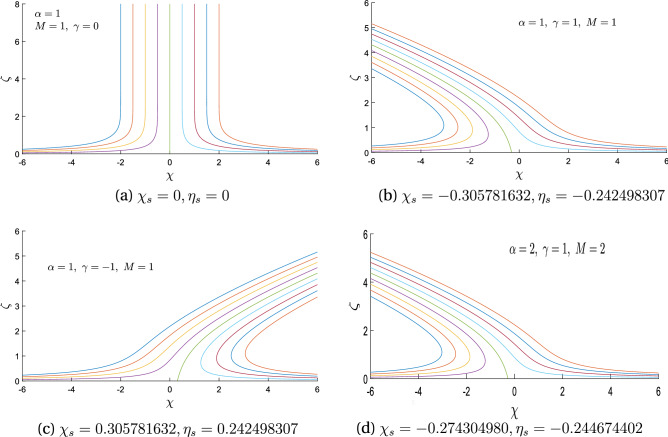
Streamline for $\alpha =1$ with the variation of $\gamma$ and M.

**Figure 6:**
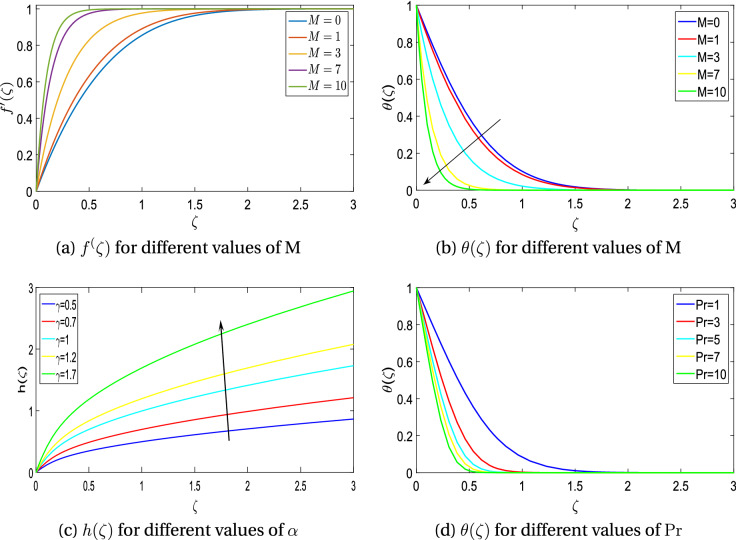
Velocity and temperature profiles for magnetic parameter *M*, rotation ratio $\alpha$ and obliqueness $\gamma$.

**Figure 7:**
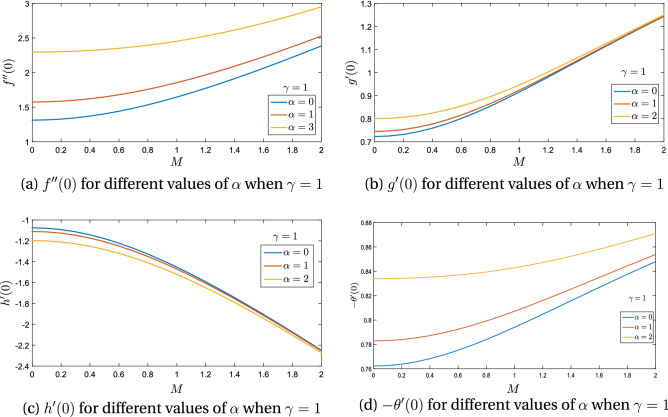
Skin friction and heat flux against magnetic parameter *M* and rotation ratio parameter $\alpha$.

We are thus confident that our farther graphical results are up to the mark. Figure 2 exhibits the streamline pattern in $\chi \eta \zeta$-plane for the case of stationary disk (i.e. in the absence of rotation). Both the cases, orthogonal ($\gamma =0$) and oblique ($\gamma \not =0$) are portrayed by varying the values of magnetic flux parameter *M*. It is noticed that this stream line pattern are towards negative $\chi$-axis for positive obliqueness and vice versa. It is further observed that point of shear center is slightly shifted due to the effect of magnet flux. For orthogonal flow, stream line patterns are uniformly distributed on the surface of the stationary disk while stream center is at the origin.

Figure 3 exhibits the streamline pattern in $\chi \eta \zeta$-plane for the case of rotating disk by assuming the obliqueness parameter $\gamma =0,-1,2$ when there is a constant magnetic flux parameter $M=2$. Disk is rotating with angular velocity $\Omega$ which is considered to be constant. This rotation of the disk in turns, generates rotation within the fluid and thereby produces a force in the radial direction of the outside called the centrifugal force. The fluid flow enter axially owing to continuity phenomena. This 3*D* flow acts as a pump; which most commonly referred to a “free disc pumping effect”.It is clear from the sub-figures that the rotational ratio approaches the stagnation point since it bears the maximum pressure. For a better understanding, stream line patterns are drawn for $\alpha =1$ in the $\chi \eta$-plane shown in Fig. 4. It is quite clear here that point of shear center is influenced by enhancing the magnetic field parameter *M*.

Figure 5 shows the streamline pattern in $\chi \zeta$-plane by varying the magnetic flux parameter *M* and rotation ratio parameter $\alpha$. The location of point of stagnation is presented in various sub-figures for some values of $\gamma$. It is very evident that when $\gamma =0$, streamlines are at right angle to $\chi$-axis and there is obliqueness for $\gamma \not =0$. Further it can also be noted that streamlines are tilted to the right for negative obliqueness and vice versa. Enhancing magnetic field parameter *M*; causes a shift in the location of the shear center. Figure 6a is plotted to explore variation due to the effect of magnetic flux parameter ‘M’ on radial velocity component $f^{\prime }({\zeta })$. Radial velocity declines with magnetic flux parameter *M*. This is because the Lorentz force is present which cause resistance within the flow. Figure 6b exhibits that thermal boundary layer thickness and temperature $\theta (\zeta )$ decreases with magnetic flux parameter *M*. Figure 6c shows that induced velocity function $h(\zeta )$ boosts with obliqueness parameter $\gamma$. Enhancement in velocity is more prominent in the vicinity of free stream. Figure 6d exhibits that temperature $\theta (\zeta )$ of the fluid and thermal boundary layer thickness significantly decreases with Prandtl number $P_r$. This results from the physical reality that a fluid with a higher Prandtl number has a low thermal diffusivity which causes a decline in temperature of the fluid. Figure 6a–d are plotted to examine the effect of magnetic flux parameter *M* and rotation ratio parameter $\alpha$ on coefficients of skin friction $(f^{\prime \prime }(0), g^{\prime }(0), h^{\prime }(0) )$ and local heat flux $(-\theta ^{\prime }(0))$.

It is quite evident from Fig. 7a,b that $f^{\prime \prime }(0)$ and $g^{\prime }(0)$ rises with magnetic flux parameter *M* and rotation ratio parameter $\alpha$. From Fig. 7c on contrary; induced component of the skin friction $h^{\prime }(\zeta )$ declines with magnetic field parameter *M* and rotation ratio parameter $\alpha$. Finally Fig. 7d shows that heat flux rate at the surface of the rotating disk enhances with an increment in Magnetic flux parameter *M* and rotation ratio parameter $\alpha$ for the case of positive obliqueness.

## Concluding remarks

The present study investigates hydromagnetic non orthogonal stagnation flow towards a rotating disk. Analysis of heat transport is also taken into account. It is worth mentioning here that the present study is relevant to spinning blades, chemical reactions, and cooling of silicon wafers. Flow control parameters’ effects such as rotation ratio parameter $\alpha$, magnetic flux parameter *M*, and obliqueness parameter $\gamma$ on heat transfer and flow properties are examined with the help of graphs and discussed in detail.The current numerical findings for the limiting situation are in very good accord with previously published literature. The investigation’s core findings can be summed up as follows:

- Radial and azimuthal coefficients of skin friction $(f^{\prime \prime }(0), g^{\prime }(0))$ significantly boosts; while induced coefficients of skin friction $h^{\prime }(0)$ declines with magnetic flux parameter *M* and rotation ratio parameter $\alpha$ for positive obliqueness.
- Momentum as well as thermal boundary layer thickness diminishes by strengthening the applied magnetic field.
- Induced velocity component $h(\eta )$ rises with obliqueness parameter $\gamma$.
- The location where the shear stress is zero, is significantly influenced with magnetic flux parameter *M* and rotation ratio parameter $\alpha$ for absolute obliqueness $|\gamma |$.
- Around the stagnation point, rotational motion is evident for the case of rotating disk.
- Rate of heat transfer at the surface of rotating disk significantly boosts with magnetic flux parameter *M* and rotation ratio parameter $\alpha$.

## Data Availability

The datasets used and/or analyzed during the current study available from the corresponding author on reasonable request.

## List of symbols

$B_{0}$: Uniform external magnetic field (Tesla)
BCs: Boundary conditions
BVP: Boundary value problem
Bi: Biot number
*c*: Specific heat
h: Induced velocity function
$h'(0)$: Tangential component of skin friction coefficient
IVP: Initial value problem
*M*: Magnetic flux parameter
ODEs: Ordinary differential equations
PDEs: Ordinary differential equations
*P*: Pressure (Pa)
$$\Pr$$: Prandtl number
*q*: Heat flux (W m$$^{-2}$$)
T: Temperature
u: Velocity component along x-axis
v: Velocity component along y-axis
z: Normal axis to the rotating disk

## Greek symbols

$\alpha$: Rotation ratio parameter
$$\gamma$$: Obliqueness parameter
$\theta$: Temperature ratio parameter
$$\nu$$: Kinematic viscosity
$$\rho$$: Density
$$\kappa$$: Thermal conductivity
$$\Omega$$: Angular velocity

## References

[1] Von Kármán T (1921). Uber laminare und turbulente reibung. Z. Angew. Math. Mech..

[2] Attia H, Aboul-Hassan A (2004). On hydromagnetic flow due to a rotating disk. Appl. Math. Model..

[3] Miklavčič M, Wang C (2004). The flow due to a rough rotating disk. Z. Angew. Math. Phys..

[4] Alam M, Hossain S, Rahman M (2016). Transient thermophoretic particle deposition on forced convective heat and mass transfer flow due to a rotating disk. Ain Shams Eng. J..

[5] Wang C (2008). Off-centered stagnation flow towards a rotating disc. Int. J. Eng. Sci..

[6] Shevchuk I, Saniei N, Yan X (2003). Impingement heat transfer over a rotating disk: Integral method. J. Thermophys. Heat Transf..

[7] Munawar S, Mehmood A, Ali A (2013). Time-dependent stagnation-point flow over rotating disk impinging oncoming flow. Appl. Math. Mech..

[8] Niimi H, Minamiyama M, Hanai S (1981). Steady axisymmetrical stagnationpoint flow impinging obliquely on a wall. J. Phys. Soc. Jpn..

[9] Rahimi A, Esmaeilpour M (2011). Axisymmetric stagnation flow obliquely impinging on a moving circular cylinder with uniform transpiration. Int. J. Numer. Methods Fluids.

[10] Sabzevar M, Rahimi A, Mozayeni H (2016). Three-dimensional unsteady stagnation-point flow and heat transfer impinging obliquely on a flat plate with transpiration. J. Appl. Fluid Mech..

[11] Bhatt RK (2012). Applications of magnetic fluid. Indian J. Eng. Mater. Sci..

[12] Turkyilmazoglu M (2012). Three dimensional MHD stagnation flow due to a stretchable rotating disk. Int. J. Heat Mass Transf..

[13] Li S, Meng W, Wang Y (2020). Numerical and experimental studies on a novel magneto-rheological fluid brake based on fluid-solid coupling. Sci. Prog..

[14] Widodo, B., Arif, D. K., Aryany, D., Asiyah, N., Widjajati, F. A. & Kamiran, K. The effect of magneto hydrodynamic nano fluid flow through porous cylinder. In *AIP Conference Proceedings*, Vol. 1867 (2017).

[15] Mustafa M (2017). MHD nanofluid flow over a rotating disk with partial slip effects: Buongiorno model. Int. J. Heat Mass Transf..

[16] Ramzan M, Chung JD, Ullah N (2017). Partial slip effect in the flow of MHD micropolar nanofluid flow due to a rotating disk-A numerical approach. Results Phys..

[17] Mahanthesh B (2018). Nonlinear radiated MHD flow of nanoliquids due to a rotating disk with irregular heat source and heat flux condition. Phys. B Condens. Matter.

[18] Soid SK, Ishak A, Pop I (2018). MHD flow and heat transfer over a radially stretching/shrinking disk. Chin. J. Phys..

[19] Reddy PS, Sreedevi P, Chamkha AJ (2017). MHD boundary layer flow, heat and mass transfer analysis over a rotating disk through porous medium saturated by Cu-water and Ag-water nanofluid with chemical reaction. Powder Technol..

[20] Gholinia M (2019). Investigation of MHD Eyring–Powell fluid flow over a rotating disk under effect of homogeneous–heterogeneous reactions. Case Stud. Therm. Eng..

[21] Zangooee MR, Hosseinzadeh K, Ganji DD (2019). Hydrothermal analysis of MHD nanofluid (TiO$$_2$$-GO) flow between two radiative stretchable rotating disks using AGM. Case Stud. Therm. Eng..

[22] Mandal S, Shit GC (2021). Entropy analysis on unsteady MHD biviscosity nanofluid flow with convective heat transfer in a permeable radiative stretchable rotating disk. Chin. J. Phys..

[23] Mehdi I, Abbas Z, Hasnain J (2022). MHD flow and heat transfer between two rotating disks under the effects of nanomaterials (MoS$$_2$$) and thermal radiation. Case Stud. Therm. Eng..

[24] Mustafa I, Javed T, Ghaffari A (2016). Heat transfer in MHD stagnation point flow of a ferrofluid over a stretchable rotating disk. J. Mol. Liq..

[25] Turkyilmazoglu M (2014). Nanofluid flow and heat transfer due to a rotating disk. Comput. Fluids.

[26] Khan W (2021). Heat transfer in steady slip flow of tangent hyperbolic fluid over the lubricated surface of a stretchable rotatory disk. Case Stud. Therm. Eng..

[27] Nayak MK (2019). Magnetohydrodynamic flow and heat transfer impact on ZnO-SAE50 nanolubricant flow over an inclined rotating disk. J. Central South Univ..

[28] Rana S, Mehmood R, Muhammad T (2021). On homogeneous–heterogeneous reactions in oblique stagnation-point flow of Jeffrey fluid involving Cattaneo–Christov heat flux. Therm. Sci..

[29] Tabassum R, Mehmood R, Malik MY (2022). Crosswise radiative convective transport of viscoplastic type nanofluid with influence of Lorentz force and viscosity variation. Arab. J. Sci. Eng..

[30] Sarkar S, Sahoo B (2021). Oblique stagnation flow towards a rotating disc. Eur. J. Mech. B/Fluids.

[31] Qasim M, Afridi MI, Wakif A, Saleem S (2019). Influence of variable transport properties on nonlinear radioactive Jeffrey fluid flow over a disk: Utilization of generalized differential quadrature method. Arab. J. Sci. Eng..

[32] Shah NA, Al-Zubaidi A, Saleem S (2021). Study of magnetohydrodynamic pulsatile blood flow through an inclined porous cylindrical tube with generalized time-nonlocal shear stress. Adv. Math. Phys..

[33] Nazeer M, Al-Zubaidi A, Hussain F, Duraihem FZ, Anila S, Saleem S (2022). Thermal transport of two-phase physiological flow of non-Newtonian fluid through an inclined channel with flexible walls. Case Stud. Therm. Eng..

[34] Gangadhar K, Edukondala Nayak R, Venkata Subba Rao M (2021). Nodal/Saddle stagnation point slip flow of an aqueous convectional magnesium oxide-gold hybrid nanofluid with viscous dissipation. Arab. J. Sci. Eng..

[35] Gangadhar K, Kumari MA, Chamkha AJ (2022). EMHD flow of radiative second-grade nanofluid over a Riga Plate due to convective heating: Revised Buongiorno’s nanofluid model. Arab. J. Sci. Eng..

[36] Gangadhar K, Kumari MA, Venkata Subba Rao M, Chamkha AJ (2022). Oldroyd-B nanoliquid flow through a triple stratified medium submerged with gyrotactic bioconvection and nonlinear radiations. Arab. J. Sci. Eng..

[37] Kotha G, Kolipaula VR, Venkata Subba Rao M, Penki S, Chamkha AJ (2020). Internal heat generation on bioconvection of an MHD nanofluid flow due to gyrotactic microorganisms. Eur. Phys. J. Plus.

[38] Gangadhar K, Bhanu Lakshmi K, Kannan T, Chamkha AJ (2022). Bioconvective magnetized Oldroyd-B nanofluid flow in the presence of Joule heating with gyrotactic microorganisms. Waves Random Complex Med..

[39] Gangadhar K, Chamkha AJ (2021). Entropy minimization on magnetized Boussinesq couple stress fluid with non-uniform heat generation. Phys. Scr..

[40] Gangadhar K, Manasa Seshakumari P, Venkata Subba Rao M, Chamkha AJ (2022). Biconvective transport of magnetized couple stress fluid over a radiative paraboloid of revolution. Proc. Inst. Mech. Eng. Part E J. Process Mech. Eng..

[41] Gangadhar K, Edukondala Nayak R, VenkataSubbaRao M, Chamkha AJ (2022). Nonlinear radiations in chemically reactive Walter’s B nanoliquid flow through a rotating cone. Proc. Inst. Mech. Eng. Part E J. Process Mech. Eng..

[42] Gangadhar K, Bhanu Lakshmi K, Venkata Subba Rao M, Chamkha AJ (2022). Thermal energy transport of radioactive nanofluid flow submerged with microorganisms with zero mass flux condition. Waves Random Complex Med..

[43] Bhargavi DN, Gangadhar K, Chamkha AJ (2022). Graphene-gold/PDMS Maxwell hybrid nanofluidic flow in a squeezed channel with linear and irregular radiations. Proc. Inst. Mech. Eng. Part E J. Process Mech. Eng..

[44] Gangadhar K, Mary Victoria E, Chamkha AJ (2022). Hydrothermal features in the swirling flow of radiated graphene-Fe$$_3$$O$$_4$$ hybrid nanofluids through a rotating cylinder with exponential space-dependent heat generation. Waves Random Complex Med..

[45] Sharma K (2022). FHD flow and heat transfer over a porous rotating disk accounting for Coriolis force along with viscous dissipation and thermal radiation. Heat Transf..

[46] Vijay N, Sharma K (2023). Dynamics of stagnation point flow of Maxwell nanofluid with combined heat and mass transfer effects: A numerical investigation. Int. Commun. Heat Mass Transf..

[47] Vijay N, Sharma K (2023). Magnetohydrodynamic hybrid nanofluid flow over a decelerating rotating disk with Soret and Dufour effects. Multidiscip. Model. Mater. Struct..

[48] Shampine, L., Gladwell, I., Shampine, L. & Thompson, S. *Solving ODEs with Matlab* (Cambridge University Press, 2003).

